# Zika Virus Outbreak in Haiti in 2014: Molecular and Clinical Data

**DOI:** 10.1371/journal.pntd.0004687

**Published:** 2016-04-25

**Authors:** John Lednicky, Valery Madsen Beau De Rochars, Maha El Badry, Julia Loeb, Taina Telisma, Sonese Chavannes, Gina Anilis, Eleonora Cella, Massimo Ciccozzi, Mohammed Rashid, Bernard Okech, Marco Salemi, J. Glenn Morris

**Affiliations:** 1 Emerging Pathogens Institute, University of Florida, Gainesville, Florida, United States of America; 2 Department of Environmental and Global Health, College of Public Health and Health Professions, University of Florida, Gainesville, Florida, United States of America; 3 Department of Health Services Research, Management, and Policy, College of Public Health and Health Professions, University of Florida, Gainesville, Florida, United States of America; 4 Christianville Foundation School Clinic, Gressier, Haiti; 5 Department of Pathology, Immunology, and Laboratory Sciences, College of Medicine, University of Florida, Gainesville, Florida, United States of America; 6 Department of Infectious Parasitic and Immunomediated Diseases, Reference Centre on Phylogeny, Molecular Epidemiology and Microbial Evolution (FEMEM)/Epidemiology Unit, Istituto Superiore di Sanita, Rome, Italy; 7 Department of Medicine, College of Medicine, University of Florida, Gainesville, Florida, United States of America; RTI International, UNITED STATES

## Abstract

**Background:**

Zika virus (ZIKV), first isolated in Uganda in 1947, is currently spreading rapidly through South America and the Caribbean. In Brazil, infection has been linked with microcephaly and other serious complications, leading to declaration of a public health emergency of international concern; however, there currently are only limited data on the virus (and its possible sources and manifestations) in the Caribbean.

**Methods:**

From May, 2014-February, 2015, in conjunction with studies of chikungunya (CHIKV) and dengue (DENV) virus infections, blood samples were collected from children in the Gressier/Leogane region of Haiti who presented to a school clinic with undifferentiated febrile illness. Samples were initially screened by RT-PCR for CHIKV and DENV, with samples negative in these assays further screened by viral culture.

**Findings:**

Of 177 samples screened, three were positive for ZIKV, confirmed by viral sequencing; DENV-1 was also identified in culture from one of the three positive case patients. Patients were from two different schools and 3 different towns, with all three cases occurring within a single week, consistent with the occurrence of an outbreak in the region. Phylogenetic analysis of known full genome viral sequences demonstrated a close relationship with ZIKV from Brazil; additional analysis of the NS5 gene, for which more sequences are currently available, showed the Haitian strains clustering within a monophyletic clade distinct from Brazilian, Puerto Rican and Guatemalan sequences, with all part of a larger clade including isolates from Easter Island. Phylogeography also clarified that at least three major African sub-lineages exist, and confirmed that the South American epidemic is most likely to have originated from an initial ZIKV introduction from French Polynesia into Easter Island, and then to the remainder of the Americas.

**Conclusions:**

ZIKV epidemics in South America, as well as in Africa, show complex dissemination patterns. The virus appears to have been circulating in Haiti prior to the first reported cases in Brazil. Factors contributing to transmission and the possible linkage of this early Haitian outbreak with microcephaly remain to be determined.

## Author Summary

Zika virus is currently spreading rapidly through the Americas, including the Caribbean, where it has emerged as a major public health problem due to the linkage with birth defects, including microcephaly. We report the isolation of Zika virus from 3 children in rural Haiti in December, 2014, as part of a study of acute undifferentiated febrile illness that was being conducted by our research group; from one of these children, we also isolated dengue virus serotype 1. On analysis of nucleotide sequence data from these and Zika strains from other locales, the South American/Haitian sequences cluster within the Asian clade and clearly branch out from a sequence circulating in Easter Island, which originated, in turn, from French Polynesia. On further analysis of one specific gene sequence for which more data were available, there appeared to be slight separation of Haitian strains and the strains from Brazil, Suriname, Puerto Rico and Guatemala, with molecular clock analysis suggesting that Zika virus was present in Haiti as early as mid-2013. These findings raise questions about the origin of Zika virus in the Caribbean, and subsequent patterns of circulation of the virus within the Americas.

## Introduction

Zika is a mosquito-borne flavivirus initially isolated in the Zika forest of Uganda in 1947 [1]. There were periodic human cases reported from Africa and Asia in the intervening decades, but it was not until 2007 that a major epidemic was reported, on Yap Island, Federated States of Micronesia [2]. Zika infections were subsequently identified in other parts of Asia, with a shift toward the Americas presaged by an outbreak on Easter Island in May, 2014 [3]. In March, 2015, cases were identified in Bahia, Brazil [4], with subsequent rapid spread through multiple Brazilian states [1,5], and other countries in South America and the Caribbean [1,5]: as of January, 2016, locally-transmitted cases had been reported by the Pan American Health Organization in Puerto Rico and 19 countries/territories in the Americas.

Infection with Zika virus (ZIKV) has traditionally been associated with asymptomatic or mild illness. Clinical manifestations, when they occur, include acute onset of fever, headache, maculopapular rash, arthralgias, myalgias, and/or non-purulent conjunctivitis [1,2]. In an outbreak in French Polynesia in 2013–14, there were, for the first time, reports of neurological and auto-immune complications, such as Guillain-Barré syndrome, in the setting of co-circulating dengue (DENV) and chikungunya (CHIKV) viruses [6,7]. With the progression of the Brazilian outbreak in 2015, the Brazilian Ministry of Health noted a striking concurrent increase in the number of infants born with microcephaly in areas with ZIKV transmission. Multiple subsequent studies have provided further documentation of the link between ZIKV and microcephaly and other birth defects, as well as with Guillain-Barré syndrome [8–14]. Based on the “strongly suspected” causal link between Zika virus and the observed fetal brain abnormalities, WHO has declared the current Zika epidemic a “public health emergency of international concern” [15].

As a step in monitoring and understanding spread of the epidemic, we report here the isolation of ZIKV from three children in Haiti in December, 2014, before the first reported Brazilian cases.

## Materials and Methods

Our group has been involved in studies of CHIKV and DENV transmission in Haiti since May, 2014, when CHIKV swept across the island of Hispaniola. Work was done in collaboration with the Christianville Foundation, which operates 4 schools in the Gressier/Leogane region of Haiti (some 20 miles west of Port-au-Prince) with a total of approximately 1,250 students from pre-kindergarten to grade 12; students attending the school receive care at no cost in an outpatient school clinic staffed by a physician and two nurses [16]. As part of these studies, UF has protocols for collection of diagnostic blood samples from children presenting to the school clinic with acute undifferentiated febrile illness (i.e., febrile illness with no localizing signs, such as would be expected with pneumonia, urinary tract infections, etc.).

Blood samples were obtained from a total of one hundred seventy-seven (n = 177) Haitian children presenting with a history of acute undifferentiated febrile illnesses between May, 2014, and February, 2015. Blood smears were prepared for microscopic analyses for malaria parasites. To obtain plasma for virologic analysis, whole blood (5 mL) was collected into purple top (K_2_EDTA) tubes (Becton, Dickinson, and Company, Franklin Lakes, New Jersey), the collected blood centrifuged to pellet red and white blood cells, and the resulting plasma transferred to sterile screw-top vials and stored at -70°C pending tests.

For the initial CHIKV and DENV screens, vRNA was extracted from virions in the plasma using a QIAamp Viral RNA Mini Kit (Qiagen Inc., Valencia, CA). The extracted vRNAs were tested using primers described by Lanciotti *et al* [17] for CHIKV and Santiago *et al* [18] for DENV types 1–4. Many specimens were positive for CHIKV and DENV1 or DENV4 vRNA (data to be presented elsewhere). Those negative for CHIKV and DENV 1–4 vRNA were also tested with a universal primer system for flavivirus: RT-PCR system Flav100F-200R [19]. No virus-specific amplicons were generated by the latter approach. Samples negative or borderline in the above assays were screened in a variety of mammalian cell lines inoculated with aliquots of plasma; detailed methods are provided in supplemental material, as are methods for transmission microscopy.

### Detection and sequencing of *Zika virus* RNA in spent cell media

As virus-specific CPE were observed in LLC-MK2 and Vero E6 cells inoculated with plasma, but the identity of the agent unknown, spent cell growth media was treated with cyanase nuclease to degrade nucleic acids external to that packaged (and thus protected) in virions using a Nucleic Acid Removal Kit (RiboSolutions, Inc., Cedar Creek, Texas), and vRNA once again extracted from the treated material using a QIAamp Viral RNA Mini Kit. A panel of PCR and RT-PCR tests were performed; for RT-PCR, first-strand synthesis was performed using random 9-mers and Accuscript High Fidelity 1st strand cDNA kit (Agilent Technologies, Santa Clara, CA). The presence of flavivirus RNA was detected using the Flav100F-200R, and *Zika virus* RNA effectively detected [20] using RT-PCR systems ZIKVF9027-ZIKVR9197c [21], 9271–9373 [22], and 835 – 911c [17]. For confirmation, PCR amplicons were purified and sequenced.

Sequencing of the complete *Zika virus* genome of one isolate (from the first patient), designated Haiti/1225/2014, was accomplished using a genome walking strategy with the PCR primers described in S1 Table. Briefly, targeted overlapping sequences (approximately 800 bp amplicons) were amplified using Accuscript High Fidelity reverse transcriptase in the presence of SUPERase-In RNase inhibitor (Ambion, Austin, TX), followed by PCR with Phusion Polymerase (New England Biolabs) with denaturation steps performed at 98°C. To obtain the 5′ and 3′ ends of the viral genome, a 5′ and 3′ system for the Rapid Amplification of cDNA Ends (RACE) was used per the manufacturer's protocols (Life Technologies, Carlsbad, CA, USA). PCR amplicons were purified, sequenced bidirectionally using Sanger Sequencing, and the sequences assembled with the aid of Sequencher DNA sequence analysis software v2.1 (Gene Codes, Ann Arbor, MI, USA). The GenBank accession number is KU509998.

### Phylogenetic analysis

All available ZIKV nucleotide sequences were downloaded from NCBI (http://www.ncbi.nlm.nih.gov/) and four data sets were assembled (S1 Table) using the following inclusion criteria: (1) sequences were published in peer-review journals; (2) known sampling time; (3) city/state was known and clearly established in the original publication. The first data set included all ZIKV complete genome sequences available in NCBI (23 sequences) and the Haiti complete genome sequence obtained in the present study. The second data set included 109 NS5 gene region reference sequences as well as NS5 sequences of the three Haitian isolates obtained in the present study. The third data set included 58 ENV gene region reference sequences as well as ENV sequences of the three Haitian isolates obtained in the present study. The fourth data set included 21 NS3 gene region reference sequences as well as the NS3 sequence of the Haitian isolate fully sequenced in the present study. Sequences in each dataset were aligned using ClustalW [23] followed by manual optimization using Bioedit [24]. The best fitting nucleotide substitution model for each data set was chosen in accordance with the results of the hierarchical likelihood ratio test (HLRT) implemented with the Modeltest software version 3.7 [25].

Detailed phylogenetic and phylodynamic methods are included in the supplemental material. In brief, the phylogenetic signal in each data set of aligned nucleotide sequences was investigated by likelihood mapping, which evaluates the tree-like signal in all possible groups of four sequences (quartets) [26];. The NS5 data set, which included the largest number of sequences and the largest number of phylogenetic informative sites (S1 Table), was used to investigate ZIKV phylogeographic patterns with the Bayesian coalescent framework implemented in Beast v 1.8 [27]. The maximum likelihood credibility (MCC) tree was chosen from the posterior distribution of trees with the TreeAnnotator program in the BEAST package. Statistical support for branching patterns in the MCC tree was obtained by calculating the posterior probability along each internal branch. The MCC tree with reconstructed ancestral states (ancestral locations inferred by Bayesian phylogeography) was manually edited in FigTree for display purposes.

### Ethics statement

The protocol for sample collection was approved by the University of Florida IRB and the Haitian National IRB. Written parental informed consent was obtained from parents or guardians of all study participants.

## Results

Zika virus was identified in plasma from three students seen in the Christianville Foundation Schools clinic. Patient #1 (described below) appears to have been infected simultaneously with DENV-1. The three case patients were from two different schools within the four-school Christianville school system; all lived in different towns/neighborhoods, within a radius of approximately 20 miles. All case patients presented within a one-week period in December, 2014. Cases of DENV-1 had been identified among children in the school clinics in the weeks before occurrence of the ZIKV cases, which, in turn, were followed by a small cluster of DENV-4 cases.

The first patient was a 15 year-old boy who presented to the clinic on December 12, 2014, with a history of subjective fever, headache, and generalized arthralgias, myalgias and asthenia. When seen, temperature was 37 degrees C, with a pulse of 92 and respiratory rate of 24, weight 51.5 Kg. He had no rash, and physical exam was unremarkable. The second patient was a 7 year-old girl who was seen on December 15, 2014 at the clinic for subjective nocturnal fever, abdominal pain, anorexia, and cough. Temperature was 37 degrees C, pulse 116, RR 28, and weight 22.8 Kg. There was no rash, and physical exam was again unremarkable. The third patient was an asymptomatic 4 year-old boy who came in December 17, 2014 for follow up after being treated for tonsillitis on November 25, when he had presented with a fever of 39 degrees C. In none of the cases would it have been possible to have identified the illness as a ZIKV infection based on clinical presentation, rather than DENV or CHIKV (and, as indicated, one child was simultaneously infected with DENV). All patients received supportive care for reported symptoms, in keeping with standard practices within the clinic.

**Fig 1:**
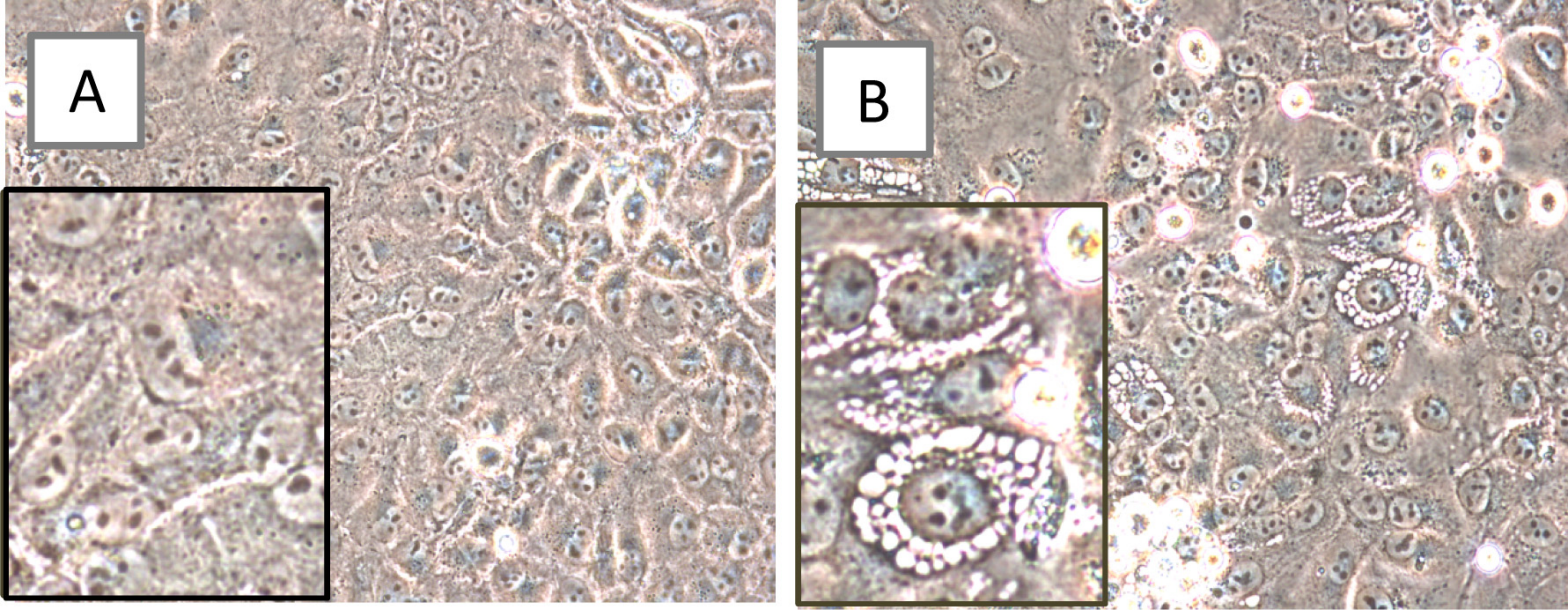
Virus-specific CPE in simian kidney cell line LLC-MK2. Non-inoculated cells (A) and cells inoculated with plasma specimen 1225/2014, 8 days post-inoculation (B). Perinuclear vacuoles are evident. Original images taken at 400x magnification; insets at approx. 800X.

**Fig 2:**
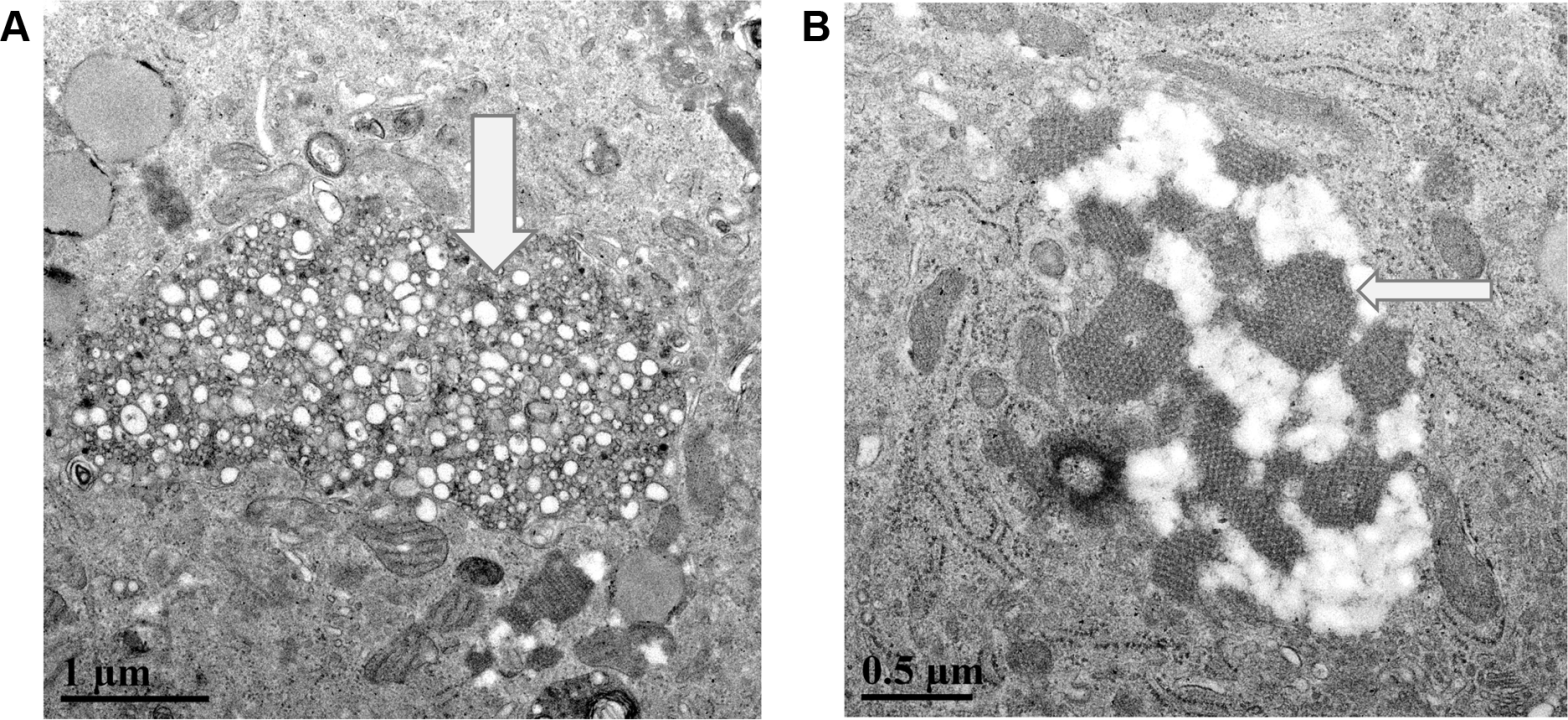
**A) Transmission electron micrograph detail of a ZIKV-infected LLC-MK2 cell.** The large arrow points out an area containing typical flavivirus-induced paracrystalline arrays/convoluted membranes in a ZIKV-infected LLC-MK2 cell. (B) Transmission electron micrograph detail of ZIKV-infected LLC-MK2 cell. Crystalline arrays of virus cores (large arrow) are shown in association with membrane vesicles.

In tissue culture, viral agents from all three patients induced subtle CPE within 4–8 days post-inoculation of human (A549, HeLa, and MRC-5) and more pronounced CPE in simian (LLC-MK2 and Vero E6) cells incubated at either 33° and 37°C. Prior to cell death, the CPE consisted of perinuclear vacuoles (Fig 1). Electron microscopy revealed features typical of flavivirus-infected cells, such as the formation of paracrystalline arrays/convoluted membranes (Fig 2A), crystalline arrays of nascent virus cores in association with double-membrane vesicles (Fig 2B), multi-membraned “whorls” (autophagosomes), individual 55–59 nm vesicles containing 40 nm virus particles, and virus particles in packets. As mentioned (Materials and Methods), direct tests of the plasma sample using RT-PCR system Flav100F-200R yielded negative results. However, specific amplicons were obtained when vRNA from cyanase-treated spent media from LLC-MK2 or Vero cells were tested with the same primers. On sequence analysis, viral agents from all three patients were identified as ZIKV.

**Fig 3:**
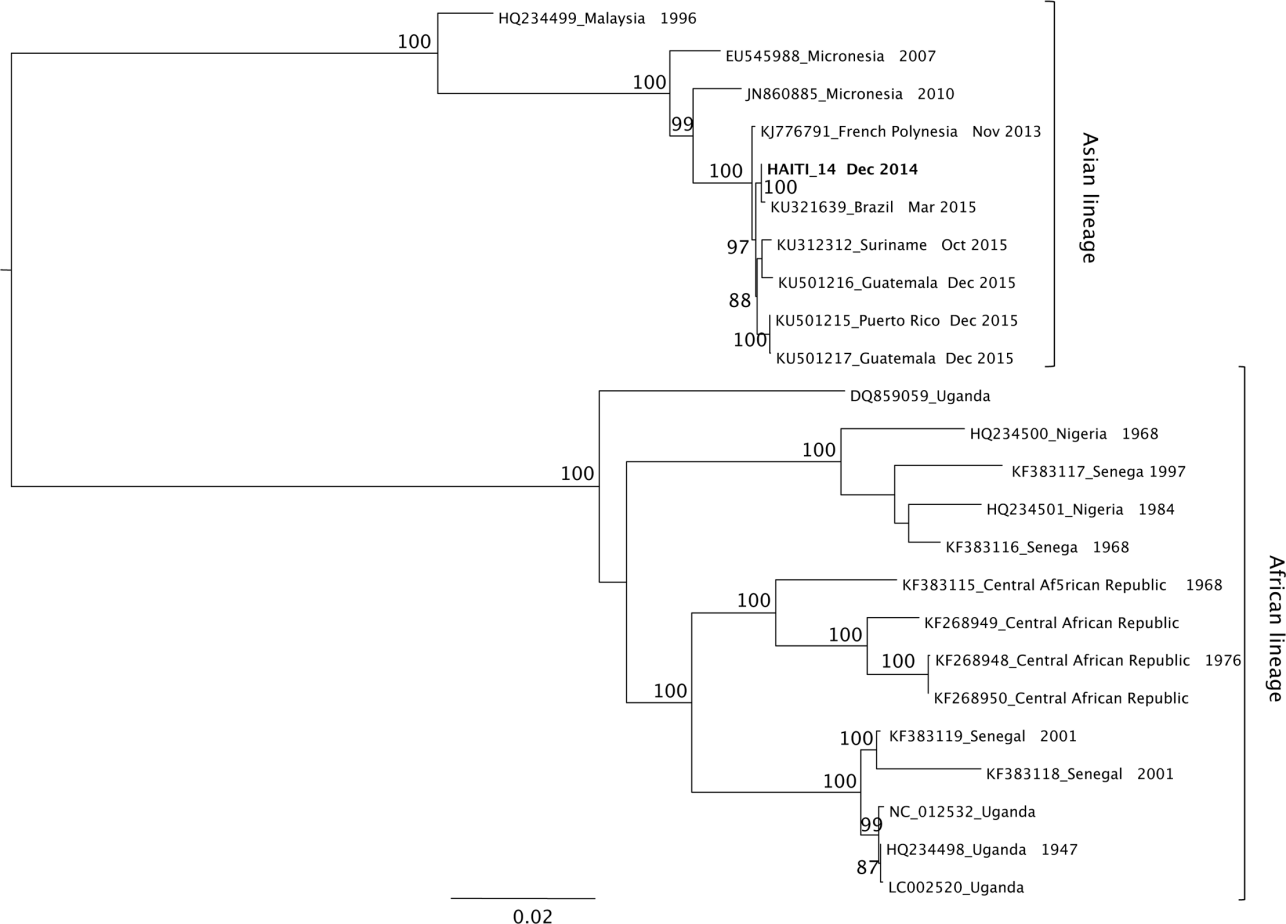
Maximum-Likelihood tree of ZIKV complete genome sequences. The tree was obtained using the best fitting nucleotide substitution model (TN93+G) selected by a hierarchical likelihood ratio test. Branches are drawn to scale in nucleotide substitutions per site according to the bar at the bottom of the tree. Percentage bootstrap (out of 1000 replicates) support values are given along branches. The Haiti sequence is in bold.

On phylogenetic analysis, likelihood mapping showed that all data sets (full genome alignment and gene-specific alignments) displayed relatively low phylogenetic noise (<20%, S2 Table) and no recombination signal was detected. The full genome alignment was, as expected, the one with the lowest phylogenetic noise (0.3%), while the NS5 alignment contained the highest number of informative sites, as well as the largest number of available sequences (S2 Table). Therefore, these two data sets were used to investigate further the phylogenetic and phylogeographic patterns of ZIKV. Maximum likelihood (ML) (Fig 3) and Neighbor-joining (NJ) (S1 Fig) trees inferred from full genome sequences consistently show two major ZIKV clades: one including African, the other one including Asian, South American and the Haitian strains. In the ML tree (Fig 3), the earliest lineage in the African clade leads to a Ugandan strain, in agreement with the scenario of ZIKV emergence in the Eastern African country [1]. Moreover, both ML and NJ trees show three highly supported monophyletic clades within the African lineage, indicating a somewhat more complex pattern than a split between West African and Nigerian strains, as recently described [17, 28]. Indeed, one clade includes Nigeria/Senegal sequences; a second one includes only Central Africa strains, while a third one includes two well-supported sub-clades, one with Ugandan and the other with Senegalese strains. South American/Haitian sequences cluster within the Asian clade and clearly branch out from a sequence circulating in Easter Island, which originated in turn from French Polynesia. The Haitian sequence clusters with a Brazilian sequence in a monophyletic clade related, in turn, to sequences from Suriname and the recently isolated strains from Guatemala and Puerto Rico [29] (Fig 3).

**Fig 4:**
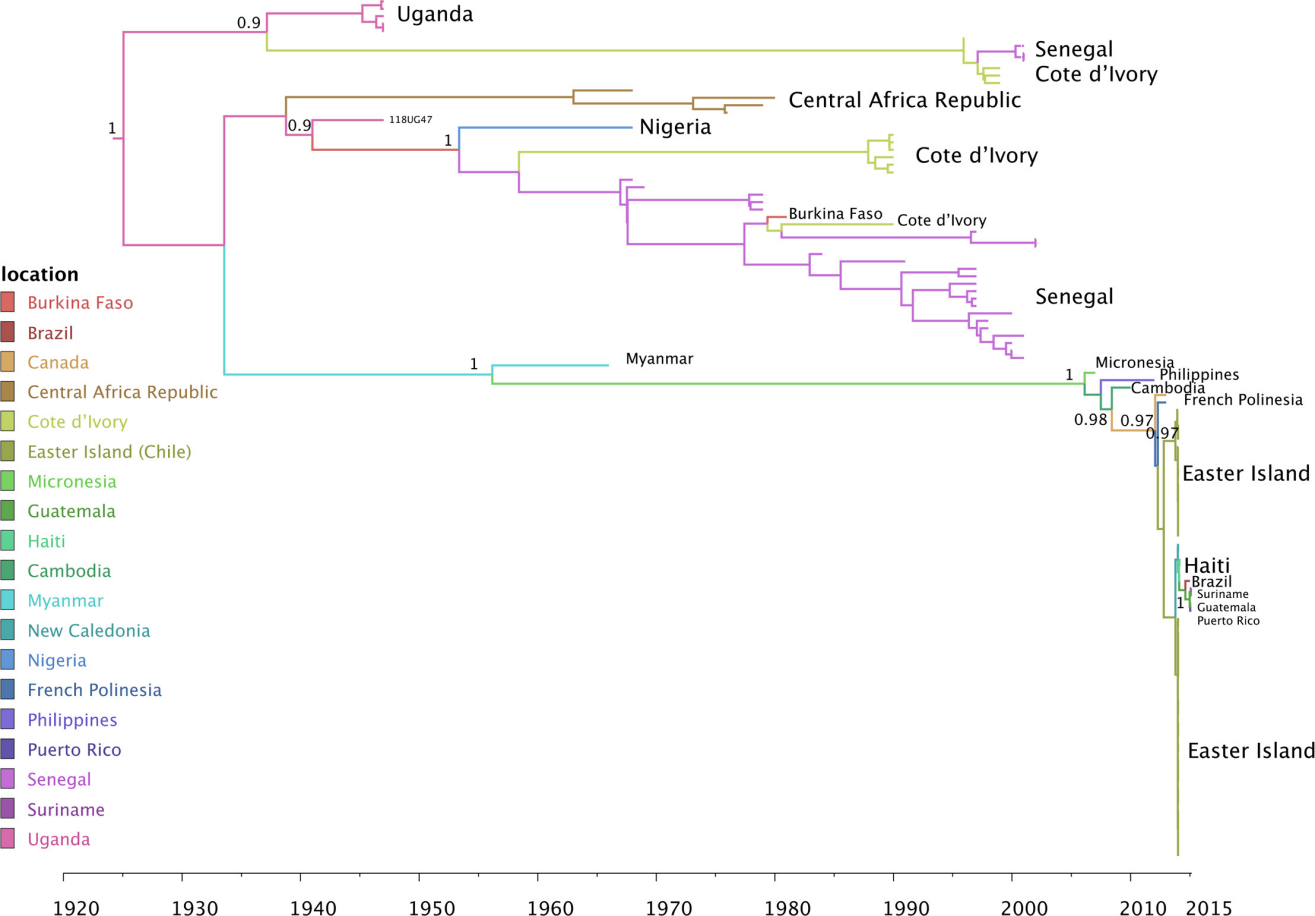
Maximum clade credibility (MCC) tree with Bayesian phylogeography reconstruction of ZIKV NS5 gene region. Branches are scaled in time and colored according to the legend to the left where each color represents the geographic location of the sampled sequence (tip branches), as well as of the ancestral lineage (internal branches) inferred by Bayesian phylogeography. The molecular clock was calibrated by using ZIKV strains known sampling times and enforcing a relaxed molecular clock with a Bayesian skyline plot demographic prior (see Supplementary Methods). For further clarity, the country of origin of the main strains in the MCC tree is also indicated to the right of each major clade (the MCC tree with full names of each isolate is provided in S2 Fig). Significant posterior probability support (p≥ 0.9) is indicated by the number along the branch. The Haitian sequences are in bold.

The pattern is confirmed by the Bayesian phylogeographic analysis showing the Asian origin of the South American sequences (Figs 4 and S2), as well as the close phylogenetic relationship between Haitian, Brazilian, Suriname and Puerto Rican strains, clustering within a larger clade of isolates from Easter Island. While not statistically significant, this latter analysis, based on the NS5 region, does show slight separation of Haitian strains and the strains from Brazil, Suriname, Puerto Rico and Guatemala. The molecular clock calibration indeed shows that the most recent common ancestor (MRCA) of the Haitian clade existed at least one year earlier (mid-2013, 95% high posterior density interval December 2012, June 2013) than the other South American lineages with the exception of the Easter Island (Chile) strains, which appear to be the oldest (Fig 4). The MRCA of the Asian lineage dates back to 1956 (95% high posterior density interval 1954–1958), while ZIKV MRCA in Africa circulated, consistently with previous estimates [27], since at least the early 1900s (95% high posterior density interval 1890–1925).

## Discussion

Our data are consistent with the occurrence of an outbreak of Zita virus infection in rural areas of Haiti west of Port-au-Prince in December of 2014. Virus was isolated from three students, coming from two different schools and different towns, suggesting that the infection was relatively widespread in the community. In keeping with prior descriptions of ZIKV infection [2], illness was mild. Two patients reported subjective fevers prior to presentation at the clinic, but were afebrile on exam (possibly due to use of local herbal antipyretics); the third patient had had a temperature of 39 degrees three weeks before (diagnosed as tonsillitis), but was asymptomatic at the time of blood collection. However, the outbreak was tightly bounded in time, with all cases occurring within a single week; we maintained similar surveillance methods across a 10 month period, and this one week was the only time that ZIKV was isolated. In keeping with reports from French Polynesia, cases occurred at a time when there was co-circulation of CHIKV and DENV, with cases immediately preceded by a cluster of DENV-1 cases (with both DENV-1 and ZIKV isolated from the first patient identified), and followed by DENV-4.

Officially, no cases of ZIKV infection were reported by the Haitian Ministry of Public Health and Population (MSPP) until January 6, 2016, when 5 cases were confirmed in patients in the metropolitan Port-au-Prince area, based on RT-PCR assays performed at the Caribbean Public Health Agency “CARPHA” Laboratory at Trinidad and Tobago. While it is difficult to assemble an accurate timeline for Zika in Haiti, given the close similarity in symptoms with DENV and CHIKV cases and their apparent co-circulation, we would hypothesize that there was an initial “wave” of ZIKV cases in the late fall of 2014 in the Leogane/Gressier region, possibly emanating from near-by Port-au-Prince. Case numbers may have been reduced by relatively low rainfall amounts at that time, with persistence in the population and, in the setting of heavy rains in the fall of 2015, occurrence of a larger epidemic in the fall of 2015/spring of 2016. Alternatively, there may have been a reintroduction of the virus in late 2015; analysis of additional sequence data, from Haiti as well as from other countries, will be necessary to reconstruct the geographic progression of strains.

Our phylogenetic analysis highlights the relative indolence of the global Zika epidemic prior to its introduction into Asia and the south Pacific in 2007. In agreement with previous reports, the virus probably emerged in Africa at the beginning of the 20^th^ century [28], where it diversified in several regional sub epidemics that, according to our analysis, span the entire equatorial Africa from Uganda, to Central Africa to Senegal. ZIKV Asian lineages, on the other hand, are of more recent origin, dating back 50–60 years ago, and the recent epidemic outbreaks in South America are probably the result of a limited introduction from French Polynesia via Easter Island no more than 3–4 year ago. The factors responsible for the rapid spread of the virus, and it’s apparent trophism for neural tissue and ability to cause severe birth defects [10–12], remain to be determined. The close association of ZIKV with the regional CHIKV epidemic, and epidemics of DENV, as we observed in Haiti, raises questions about immunologic interactions among these viruses, and/or the possibility that co-infection facilitates viral transmission or severity. Our observations highlight the critical ongoing need for careful epidemiologic and basic science research to guide public health interventions in Haiti and elsewhere where ZIKV is now epidemic.

## Supporting Information

S1 Text Supplemental Material. (DOCX)

S1 Fig Neighbor joining tree of ZIKV complete genome sequences. The tree was obtained using the best fitting nucleotide substitution model (TN93+G) selected by a hierarchical likelihood ratio test. Branches are drawn to scale in nucleotide substitutions per site according to the bar at the bottom of the tree. Significant posterior probability support (p≥ 0.9) is indicated by the number along the branch. The Haiti sequence is in bold.
(PDF)

S2 Fig Maximum clade credibility (MCC) tree with Bayesian phylogeography reconstruction of ZIKV NS5 gene region and tips labeled according to strain names. Month (when available) and year of isolation are also indicated for each strain.
(PDF)

S1 Table Primers for sequencing of ZIKV Haiti/1/2014. (DOCX)

S2 Table ZIKV evolutionary model and phylogenetic signal in different genes. (DOCX)

S3 Table Marginal likelihood estimates and Bayes factors comparing molecular clock and demographic models inferred by Bayesian phylogenetics of ZIKV NS5 gene sequences. (DOCX)
