# Supplementary material for: Zika Virus Outbreak in Haiti in 2014: Molecular and Clinical Data
Source: PLoS Negl Trop Dis. 2016 Apr 25;10(4):e0004687. doi: 10.1371/journal.pntd.0004687 (PMC4844159; doi:10.1371/journal.pntd.0004687)
Supplement: S1 Text — (DOCX) [file pntd.0004687.s001.docx]

**SUPPLEMENTAL MATERIAL**

**Detailed Methods, Lednicky *et al***

**Tissue Culture and Transmission Microscopy:** Cell lines A549 (CCL-185), BHK-21 (CCL-10), HeLa (CCL-2), LLC-MK2 (CCL-7), MDCK, (CCL-34), MRC-5 (CCL-171) and Vero E6 (CRL-1586), obtained from the American Type Culture Collection (ATCC) (Manassas, VA, USA), were propagated as monolayers at 37°C and 5% CO_2_ in Advanced Dulbecco's Modified Eagle's Medium (aDMEM) or Eagle's Minimal Essential Medium (EMEM) (Invitrogen, Carlsbad, CA, USA), as appropriate per cell line. aDMEM and EMEM were supplemented with 2 mM L-Alanyl-L-Glutamine (GlutaMAX, Invitrogen, Carlsbad, CA, USA.), antibiotics [PSN; 50 µg/ml penicillin, 50 µg/ml streptomycin, 100 µg/ml neomycin (Invitrogen, Carlsbad, CA, USA)], and 10% (v/v) low IgG, heat-inactivated gamma-irradiated fetal bovine serum (HyClone, Logan, UT, USA). EMEM was also supplemented with sodium pyruvate (Invitrogen Corp.) and non-essential amino acids (Hyclone, Logan, UT, USA). No mosquito cell lines were used for this work. Cell cultures were grown in T25 cell culture flasks (cell growth surface of 25-cm^2^) to 80% confluency. For each sample, duplicate cultures were prepared, and one set incubated at 37°C, the other at 33°C. The inoculated cells were observed daily for signs of cytopathic effects (CPE), with re-feeds performed every 3 days. The cultures were maintained for one month before being considered negative for virus isolation. Non-infected cells were maintained and re-fed in parallel for comparison.

Transmission electron microscopy was performed at the University of Florida Interdisciplinary Center for Biotechnology Research. Cells were fixed in 4% paraformaldehyde, 2% glutaraldehyde in 0.1M cacodylate buffer, pH 7.24, embedded in EMbed812/Araldite epoxy resin, sectioned  into 100nm sections, stained with 2% uranyl acetate and Reynold’s lead citrate, and viewed using an FEI Tecnai Spirit transmission electron microscope.

**Phylogenetic/Phylodynamic Analysis:** The phylogenetic signal in each data set of aligned nucleotide sequences was investigated by likelihood mapping, which evaluates the tree-like signal in all possible groups of four sequences (quartets) [1]. For each quartet, the likelihoods of the three possible unrooted trees are estimated and reported as a dot in an equilateral triangle (the likelihood map). The distribution of the dots in the likelihood map provide information on the phylogenetic signal in the data sets: the three corners represent fully resolved tree topologies, i.e. the presence of treelike phylogenetic signal in the data; the center represents the star-like phylogeny, i.e. phylogenetic noise, and the three side areas indicate network-like phylogeny, i.e. presence of recombination or conflicting phylogenetic signals. Findings from extensive simulation studies show that the central area with < 30% dots indicate robust phylogenetic signal. The likelihood mapping analysis was performed with the program TREE-PUZZLE [1]. The phylogenetic signal was also investigated by counting the number of Parsimony Informative Sites. Neighbor-joining (NJ) and maximum likelihood (ML) phylogenetic trees were estimated using the best fitting nucleotide substitution model and statistical robustness of the branching order within the tree were assessed by bootstrapping (1000 replicates) and fast likelihood-based SH-like probability test. Calculations were carried out with the IQTREE software [2].

The NS5 data set, which included the largest number of sequences as well as informative sites (Supplementary Table S1), was used to investigate ZIKV phylogeographic patterns with the Bayesian coalescent framework implemented in Beast v 1.8 [4]. Three different coalescent priors were evaluated by enforcing either a strict or relaxed molecular clock: constant population size and two non-parametric models of population change (Bayesian Gaussian Markov Random Field skyride plot and Bayesian skyline plot). For each model a Markov Chain Monte Carlo (MCMC) was run 100 to 500 million generations, sampling every 10,000 steps. Proper mixing of the MCMC was assessed on the basis of the effective sampling size (ESS). The MCMC was halted when each estimated parameter had an ESS values > 250. Models were compared pair-wise by calculating the Bayes factor with marginal likelihoods estimated *via* path sampling and stepping stone methods implemented in BEAST [5]. The selected molecular clock/demographic model (relaxed clock with Bayesian Skyline Plot coalescent prior, see Supplementary Table S2) was then used for the Bayesian phylogeographic analysis based on the continuous time Markov Chain (CTMC) process over discrete sampling locations, with Bayesian Stochastic Search Variable Selection (BSSVS), implemented in BEAST 1.8 [6]. An MCMC was run 100 million generations, sampling every 10,000 steps and proper mixing assessed by evaluating the ESS as described above. The maximum likelihood credibility (MCC) tree was chosen from the posterior distribution of trees with the TreeAnnotator program in the BEAST 1.8 package. Statistical support for branching patterns in the MCC tree was obtained by calculating the posterior probability along each internal branch. The MCC tree with reconstructed ancestral states (ancestral locations inferred by Bayesian phylogeography) was manually edited in FigTree (http://tree.bio.ed.ac.uk/software/figtree/) for display purposes.

*References*

1. Strimmer K, von Haeseler A.1997. Likelihood-mapping: a simple method to visualize phylogenetic content of a sequence alignment. Proc Natl Acad Sci U S A. 94(13):6815-9
2. Schmidt HA, Strimmer K, Vingron M, von Haeseler A. 2002. TREE-PUZZLE: maximum likelihood phylogenetic analysis using quartets and parallel computing. Bioinformatics 18(3):502-4.
3. Nguyen LT, Schmidt HA, von Haeseler A, Minh BQ. 2015. IQ-TREE: a fast and effective stochastic algorithm for estimating maximum-likelihood phylogenies. Mol Biol Evol. 32(1):268-74.
4. Drummond AJ, Suchard MA, Xie D, Rambaut A. 2012. Bayesian phylogenetics with BEAUti and the BEAST 1.7. Mol Biol Evol. 29(8):1969-73.
5. Baele G, Li WL, Drummond AJ, Suchard MA, Lemey P. 2013. Accurate model selection of relaxed molecular clocks in Bayesian phylogenetics. Mol Biol Evol. 30(2):239-43.
6. Lemey P, Rambaut A, Drummond AJ, Suchard MA. 2009. Bayesian phylogeography finds its roots. PLoS Comput Biol. 5(9):e1000520.
