# Supplementary figures and images for: Zika Virus Outbreak in Haiti in 2014: Molecular and Clinical Data
Source: PLoS Negl Trop Dis. 2016 Apr 25;10(4):e0004687. doi: 10.1371/journal.pntd.0004687 (PMC4844159; doi:10.1371/journal.pntd.0004687)

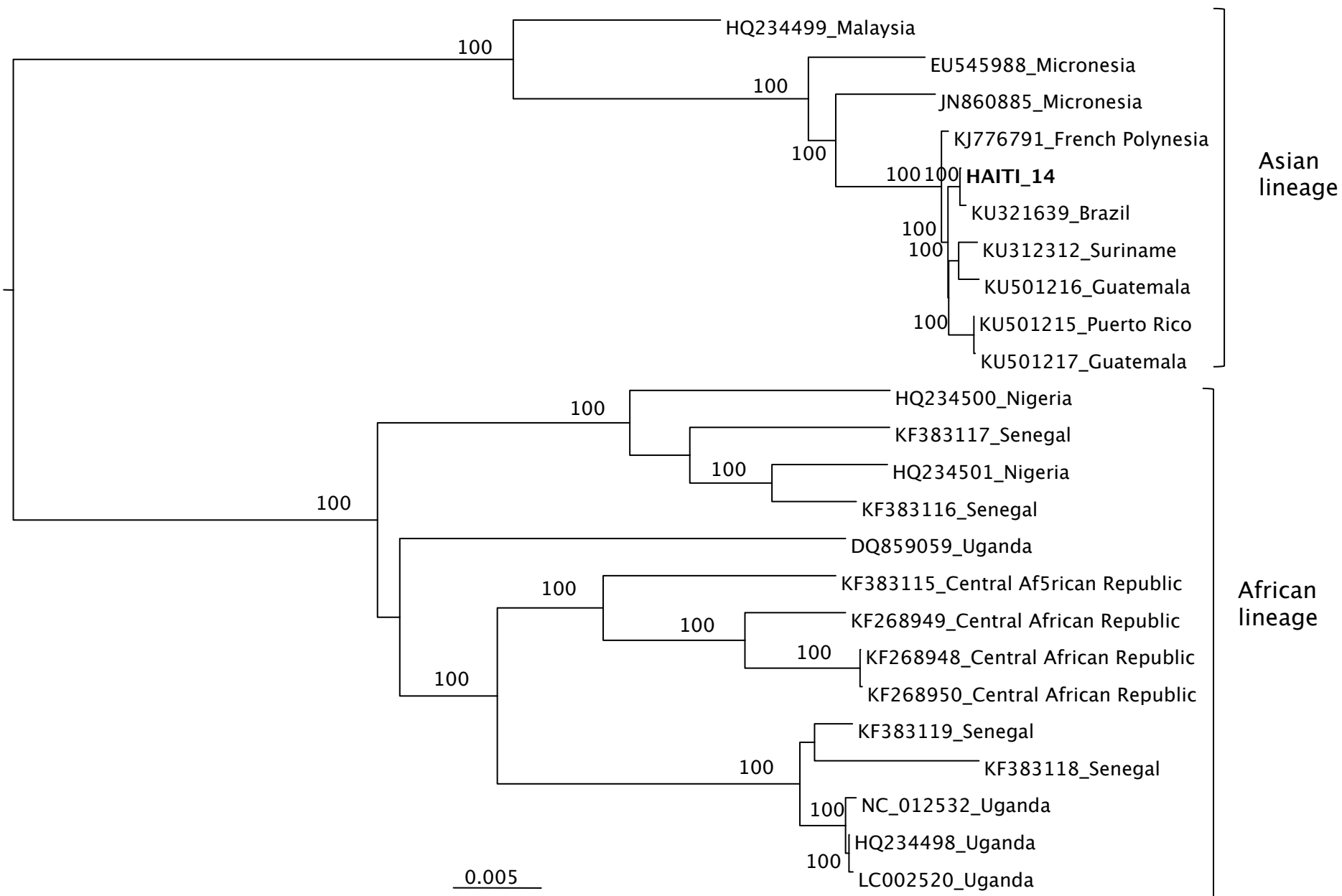

Supplement: S1 Fig — The tree was obtained using the best fitting nucleotide substitution model (TN93+G) selected by a hierarchical likelihood ratio test. Branches are drawn to scale in nucleotide substitutions per site according to the bar at the bottom of the tree. Significant posterior probability support (p≥ 0.9) is indicated by the number along the branch. The Haiti sequence is in bold. (PDF) [file pntd.0004687.s002.pdf]
