# Supplementary material for: Zika Virus Outbreak in Haiti in 2014: Molecular and Clinical Data
Source: PLoS Negl Trop Dis. 2016 Apr 25;10(4):e0004687. doi: 10.1371/journal.pntd.0004687 (PMC4844159; doi:10.1371/journal.pntd.0004687)
Supplement: S1 Table — (DOCX) [file pntd.0004687.s004.docx]

**Supplementary Table S1. Primers for sequencing of ZIKV Haiti/1/2014**.

| **Primer** | **Sequence (5’ -3’)** | **Location in KU509998.2** |
| --- | --- | --- |
| 5’ UTR –R1 | catattgacaatccggaatcctcc | 154 - 131 |
| ZIKV-F1 | ATGAAAAACCCAAAAAAGAAATCC | 107 - 130 |
| ZIKV-R1 | caagcgatggcagctgctgctaac | 903 - 880 |
| ZIKV-F2 | gaatacacaaagcacttgattagagtc | 821 - 847 |
| ZIKV-R2 | gaaccactccttgtgaaccaaccagtg | 1630 - 1604 |
| ZIKV-F3 | cttgattgtgaaccgaggacagg | 1538 - 1560 |
| ZIKV-R3 | tccaaacaatgatttgaaagctgctc | 2350 - 2325 |
| ZIKV-F3A | tggaagcctaggacttgattgtgaac | 1525 - 1550 |
| ZIKV-F4 | ctcattgggcaagggcatccatc | 2293 - 2315 |
| ZIKV-R4 | ccagtagcctagatcactgtgtac | 3091 - 3068 |
| ZIKV-F5 | ggaacagctgttaagggaaaggag | 3041 - 3064 |
| ZIKV-R5 | ccaattagctctgaagatgaaagatac | 3841 - 3815 |
| ZIKV-F6 | cattcaaagtcagaccagcgttgc | 3789 - 3812 |
| ZIKV-R6 | gcaccactcctttttccagtcttga | 4620 - 4598 |
| ZIKV-F7 | gcagctggagcgtggtacgtatacg | 4571 - 4595 |
| ZIKV-R7 | gagtgggtgacattgactgctgttg | 5370 - 5346 |
| ZIKV-F8 | gcccttagagggcttccagtgcgttatatg | 5315 - 5344 |
| ZIKV-R8 | gaggccatcttggaggtaaatattg | 6121 - 6097 |
| ZIKV-F9 | cacactggcttgaagcaagaatgct | 6066 - 6090 |
| ZIKV-R9 | gccatttggttgtcctggggagatctttg | 6867 - 6839 |
| ZIKV-F10 | ggtggtgctcatacctgagccag | 6811 - 6833 |
| ZIKV-R10 | ccaagtaacttcccctaaaaatgttacac | 7610 - 7582 |
| ZIKV-F11 | ctggaactcctctacagccacttcac | 7555 - 7580 |
| ZIKV-R11 | gtggtggacacactttttatggtgttg | 8373-8347 |
| ZIKV-F12 | cccgcaactctacacatgagatgtac | 8301 - 8326 |
| ZIKV-R12 | ctagccacatataccagatggcgc | 9107 - 9084 |
| ZIKV-F13 | gaatttggaaaggccaagggcag | 9059 - 9081 |
| ZIKV-R13 | ggtggcggcagggaaccacaatg | 9863 - 9841 |
| ZIKV-F14 | ctccatctcaaggacgggaggtc | 9818 - 9840 |
| ZIKV-R14 | gcgcgtggggttttttgactcagtg | 10564 - 10540 |
| ZIKV-F15 | catgctgcctgtgagcccctcagaggac | 10513 - 10540 |
| ZIKV-R15 | ccactagtccctcttctggagatcc | 10667 - 10643 |
| 3’ UTR – F1 | ctacctatccacccaagttcgctac | 10318 - 10342 |
| 3’ UTR – F2 | gtggcgaccttccccacccttcaat | 10591 - 10615 |
