# Supplementary material for: Zika Virus Outbreak in Haiti in 2014: Molecular and Clinical Data
Source: PLoS Negl Trop Dis. 2016 Apr 25;10(4):e0004687. doi: 10.1371/journal.pntd.0004687 (PMC4844159; doi:10.1371/journal.pntd.0004687)
Supplement: S2 Table — (DOCX) [file pntd.0004687.s005.docx]

**Supplementary Table S2. ZIKV evolutionary model and phylogenetic signal in different genes**

| GENE | N# SEQUENCES* | NT LENGHT | EVOLUTIONARY  MODEL | COSTANT  SITES | PI sites** | PHYLOGENETIC NOISE*** |
| --- | --- | --- | --- | --- | --- | --- |
| ENV | 61 | 282 | T92+G | 65.25% | 74 (26.2%) | 10.5% |
| NS5 | 112 | 531 | TN93+G | 65.91% | 147 (27.7%) | 18.2% |
| NS3 | 22 | 771 | TN93+G | 75.36% | 130 (16.8%) | 2.8% |
| FULL GENOME | 24 | 10269 | TN93+G | 78.04% | 1742 (17.0%) | 0.3% |

* Sequences currently available (including the new Haitian isolates) in GenBank.

** Number of Parsimony Informative sites

*** Star-like signal (proportion of dots in the center of the likelihood map) inferred by likelihood mapping analysis
