# Supplementary material for: Zika Virus Outbreak in Haiti in 2014: Molecular and Clinical Data
Source: PLoS Negl Trop Dis. 2016 Apr 25;10(4):e0004687. doi: 10.1371/journal.pntd.0004687 (PMC4844159; doi:10.1371/journal.pntd.0004687)
Supplement: S3 Table — (DOCX) [file pntd.0004687.s006.docx]

**Supplementary Table S3. Marginal likelihood estimates and Bayes factors comparing molecular clock and demographic models inferred by Bayesian phylogenetics of ZIKV NS5 gene sequences**

| Model^1^ | PS^2^ | SS^3^ |  | PS lnBF(SC/RC)^4^ | SS lnBF(SC/RC)^5^ |  | RC^6^ | PS lnBF^7^ | SS lnBF^8^ |
| --- | --- | --- | --- | --- | --- | --- | --- | --- | --- |
| RC_cost | -2700.0 | -2688,7 |  | 63.5 | 75.6 |  | **BSP**-cost | 15.7 | 3.6 |
| SC_cost | -2763,5 | -2764,3 |  |  |  |  |  |  |  |
|  |  |  |  |  |  |  |  |  |  |
| **RC_BSP** | **-2684,3** | **-2685,1** |  | **37.3** | **37.5** |  | **Cost**-Skygrid | 47.1 | 59.3 |
| SC_BSP | -2721,6 | -2722,6 |  |  |  |  |  |  |  |
|  |  |  |  |  |  |  |  |  |  |
| RC_Skygrid | -2747,1 | -2748,0 |  | 84.1 | 84.2 |  | **BSP**-Skygrid | 62.8 | 62.9 |
| SC_Skygrid | -2831,2 | -2832,2 |  |  |  |  |  |  |  |

1. Bayesian molecular clock and demographic models used in the analysis: SC = strict molecular clock; RC = relaxed molecular; cost = constant population size; BSP = Bayesian Skyline Plot; Skygrid = Bayesian skygrid plot.
2. Model’s Marginal likelihood estimate using to the Path Sampling (PS) method.
3. Model’s Marginal likelihood estimate using to the Stepping Stone (SS) method.
4. Bayes Factor comparing strict (SC) versus relax (RC) molecular clock model based on PS estimates of marginal likelihoods.
5. Bayes Factor comparing strict (SC) versus relax (RC) molecular clock model based on SS estimates of marginal likelihoods.
6. Demographic models (enforcing a relaxed molecular clock) compared.
7. Bayes Factor comparing different demographic models based on PS estimates of marginal likelihoods.
8. Bayes Factor comparing different demographic models based on SS estimates of marginal likelihoods.

Selected models (according the BF comparisons) are given in bold.
