# Supplementary material for: Zika Virus Outbreak in Haiti in 2014: Molecular and Clinical Data
Source: PLoS Negl Trop Dis. 2016 Apr 25;10(4):e0004687. doi: 10.1371/journal.pntd.0004687 (PMC4844159; doi:10.1371/journal.pntd.0004687)
Supplement: S2 Fig — Month (when available) and year of isolation are also indicated for each strain. (PDF) [file pntd.0004687.s003.pdf]

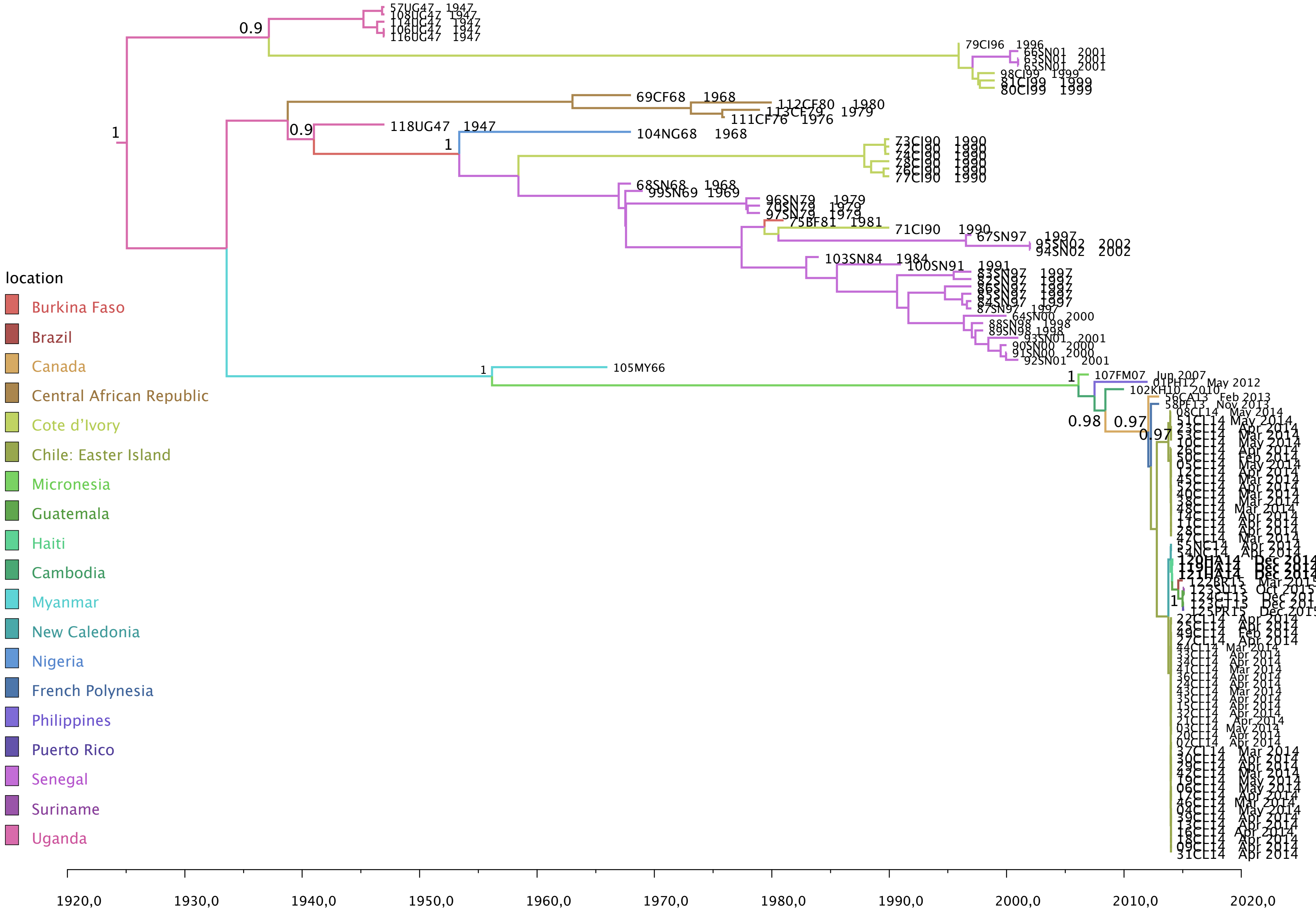

location
Burkina Faso
Brazil
Canada
Central African Republic
Cote d'Ivory
Chile: Easter Island
Micronesia
Guatemala
Haiti
Cambodia
Myanmar
New Caledonia
Nigeria
French Polynesia
Philippines
Puerto Rico
Senegal
Suriname
Uganda
1920,0
1930,0
1940,0
1950,0
1960,0
1970,0
1980,0
1990,0
2000,0
2010,0
2020,0
1
0.9
0.9
1
1
1
0.98
0.97
0.97
57UG47 1947
116UG47 1947
79CI96 1996
80CI99 1999
69CF68 1968
112CF80 1980
111CF76 1976
118UG47 1947
104NG68 1968
73CI90 1990
77CI90 1990
68SN68 1968
99SN69 1969
96SN79 1979
75BF81 1981
71CI90 1990
67SN97 1997
94SN02 2002
103SN84 1984
100SN91 1991
64SN00 2000
92SN01 2001
105MY66
107FM07 Jun 2007
56CA13 Feb 2013
58PF13 Nov 2013
08CL14 May 2014
31CL14 Apr 2014
